# Piglets Learn to Use Combined Human-Given Visual and Auditory Signals to Find a Hidden Reward in an Object Choice Task

**DOI:** 10.1371/journal.pone.0164988

**Published:** 2016-10-28

**Authors:** Sandy Bensoussan, Maude Cornil, Marie-Christine Meunier-Salaün, Céline Tallet

**Affiliations:** PEGASE, Agrocampus Ouest, INRA, Saint-Gilles, France; University of Portsmouth, UNITED KINGDOM

## Abstract

Although animals rarely use only one sense to communicate, few studies have investigated the use of combinations of different signals between animals and humans. This study assessed for the first time the spontaneous reactions of piglets to human pointing gestures and voice in an object-choice task with a reward. Piglets (*Sus scrofa domestica*) mainly use auditory signals–individually or in combination with other signals—to communicate with their conspecifics. Their wide hearing range (42 Hz to 40.5 kHz) fits the range of human vocalisations (40 Hz to 1.5 kHz), which may induce sensitivity to the human voice. However, only their ability to use visual signals from humans, especially pointing gestures, has been assessed to date. The current study investigated the effects of signal type (visual, auditory and combined visual and auditory) and piglet experience on the piglets’ ability to locate a hidden food reward over successive tests. Piglets did not find the hidden reward at first presentation, regardless of the signal type given. However, they subsequently learned to use a combination of auditory and visual signals (human voice and static or dynamic pointing gestures) to successfully locate the reward in later tests. This learning process may result either from repeated presentations of the combination of static gestures and auditory signals over successive tests, or from transitioning from static to dynamic pointing gestures, again over successive tests. Furthermore, piglets increased their chance of locating the reward either if they did not go straight to a bowl after entering the test area or if they stared at the experimenter before visiting it. Piglets were not able to use the voice direction alone, indicating that a combination of signals (pointing and voice direction) is necessary. Improving our communication with animals requires adapting to their individual sensitivity to human-given signals.

## Introduction

Communication between two individuals—a sender and a receiver—requires the use of their senses to convey signals containing non-permanent information which then alters the receiver’s behaviour [1,2]. Between humans, these signals are mainly auditory (verbal and non-verbal), visual, and tactile [3]. Animal communication depends on their Umwelt [4], that is on the stimuli species can perceive with their sensory organs. Communication also occurs between animals and humans: human-given signals can entirely or partially fit in an animal’s umwelt. However misinterpretation occurs when perceptions do not match emissions [5]. Humans and animals living in the same environment have to adapt to each other, coping with differences in/the limitations of what they can convey and perceive. Understanding animal perception of human-given signals is necessary to improve interspecific communication [3,5].

The ability of animals to discriminate between people has been regularly utilized to test their perception of human signals. Some species can rely on only one form of sensorial information, e.g. California sea lions (*Zalophus californianus*) [6] can identify specific people using only voice recordings. Others may rely on combinations of signals: ewes (*Ovis aries*) [7] and cats (*Felis silvestris catus*) [8] both succeeded in a similar task using combinations of visual, olfactory and auditory information. This ability varies among species: humans can be identified by their faces by Guinea baboons (*Papio papio*) [9] and cows (*Bos taurus*) [10], and by their odor and voices by dogs (*Canis lupus familiaris*) [11] and African elephants (*Loxodonta Africana*) [12] respectively.

Some wild and domesticated animals are sensitive to human signals and the evolution of such skills/abilities is still under investigation. Some domesticated animals (e.g. cats, dogs, horses, pigs) are able to use humans’ signals in cognitive tasks [13]. The potential impact of domestication on these communicative skills has long been highlighted but is regularly questioned [13,14,15,16] especially because some wild species also use human signals. Cross-species comparisons are therefore necessary in order to understand the mechanisms underlying these communicative capacities which may have evolved differently in different species [13]. One hypothesis is that animal sensitivity to human signals may have evolved due to regular interactions with humans in situations necessitating communicative and cooperative skills [16]. For instance, dogs (*Canis lupus familiaris*) are highly sensitive to human signals [16, 17]: they are able to follow human pointing gestures [5] and vocal orders [8] used in hunting, herding and companioning. Another hypothesis is that sensitivity to human signals may, at a certain level, be a consequence of the selective breeding of animals for enhanced adaptability [16]. Increased tameness with reduced fear and aggression would be inseparable from enhancing cognitive skills. This has been observed in silver foxes (*Vulpes vulpes*), experimentally domesticated for 45 years, which were better at using pointing gestures than foxes not selected for tameness [18]. These processes are still studied to determine the responses of animals to signals used in referential communication, about an object for example, including a sender utilising one of the animal’s senses.

Previous research regarding object choice tasks has mainly involved the use of visual rather than auditory or olfactory signals, probably in order to reduce logistical difficulties. For instance visual signals were studied in dogs [19], horses (*Equus caballus*) [20], goats (*Capra Hircus*) [21], wolves (*Canus lupus lupus*) [22], dolphins (*Tursiops truncatus*) [23], seals (Arctocephalus pusillus) [24], wild ferret hybrids (*Mustela*) [25], African elephants [26], chimpanzees (*Pan troglodytes*) [27] and gorillas (*Gorilla gorilla*) [15,28]. To our knowledge however studies on auditory signals appear less numerous; in dogs (voice direction [29]) and elephants (referential word for the location [30] and sound made by the reward itself [31]). Olfactory signals have been tested even less although we can site the work on elephants [31]. The sensitivity of animals to human signals probably involves species-specific sensory preferences, even if learning processes are also involved. Covering a wider sensitivity range, combinations of signals can also be simultaneously transferred, providing complementary forms of information [5, 32]. Therefore, more knowledge is needed regarding the sensitivity of animals to human-given signals, expressed both alone and in combination especially on less studied signals.

According to Seabrook [33], people interacting with animals use complex combinations of individual factors such as sound, smell or gesture which are difficult to study separately. Although few studies have been conducted on this topic, animals appear to use combinations of information forms. Tanida and Nagano [34] found that piglets discriminated between handlers more easily when visual, auditory and olfactory information were combined, than when one or more factors were obstructed. When observed in an object-choice task, chimpanzees performed better when locating hidden food with a combination of human visual and auditory signals than when only one signal was given [27]. This supports the theory that combinations of signals might be more informative than individual ones, although further investigations are still required.

Pigs are a suitable model to study interspecific communication, in view of their long history of domestication. Dogs were the first animals to be domesticated around 15,000 years ago, while pig domestication is estimated to have begun 9,000 years ago. Data on pigs indicate a hybrid origin of some major “European” pig breeds, and provide strong evidence that the ancestral subspecies of wild boar in Europe and Asia were genetically distinct. Divergence of the ancestral boar subspecies is estimated to have been 500,000 years ago, well before domestication [35]. Pigs have a broad range of social skills which involve various sensorial pathways. Hearing is well developed in pigs, with a large auditory spectrum ranging from 42 Hz to 40.5 kHz [36], into which human vocal productions fit (80 Hz to 1,4 kHz) [37]. Acoustic signals are various and of major importance in the social behaviour of pigs [38] and experienced handlers suggest that pigs respond to human voices and their variations [39]. The effects of human auditory signals were studied to assess the ability of piglets to discriminate between familiar handlers [34]. Determining the sensitivity of piglets to these signals could help understand the impact of human interactions while working with animals. The mechanisation of modern facilities has reduced the time spent with animals to minimal interventions [40] which are frequently aversive in piglets (tooth resection, vaccines…) and can lead to the appearance of undesirable behaviours (escape, screaming…). Increasing our understanding of the effects of human communicative signalling will improve our ability to interact with animals, increasing both animal and handler welfare, while also producing economic gains.

Evidence supporting the use of human visual signals by pigs is inconsistent in the literature [41, 42]. Albiach-Serrano et al. [41] determined that pigs submitted to an object-choice task could not find a hidden reward with a pointing gesture whereas captive wild boars could. These differences were explained by the stimuli that the subjects had previously encountered: the wild boars had been exposed to the public pointing at food thrown in their enclosure while pigs had not. The effects of previous exposure to relevant stimuli could also explain the results reported in Nawroth et al. [42] in which piglets were able to use pointing gestures. Nawroth et al. [42] reported a successful training criterion to be the ability of piglets to go straight to the container which the experimenter extended their arm towards (and which contained the reward). Similarities between this training gesture and the dynamic pointing gestures tested subsequently cannot prevent anyone from questioning the excellent reported results. Differences in the ages/physiological states of the experimental animals in these studies (respectively sub-adult and adult pigs vs piglets) may by themselves explain the inconsistent results, as could age-/breed-related learning abilities and experience acquired before the tests.

Some species which would not normally use spontaneous human-given signals are able to learn to use them. This is the case for Clark’s nutcrackers (*Nucifraga columbiana*) [43], which learnt to use gaze orientation to retrieve food in an object-choice task after several sessions. Olive baboons (*Papio anubis*) also performed begging gestures to obtain a reward from an experimenter even though they did not belong to the group trained to use this gesture. Piglets are known to be good learners in tasks associated with food delivery [44] and are able to learn through the course of experiments [45]. However, the spontaneous use of human pointing gestures and/or auditory signals reported in piglets, as well the effect of experience on their response still need attention.

To our knowledge, this study is the first to assess the spontaneous reactions of piglets to human visual signals and to human voice in an object-choice task with a reward. We submitted piglets to various signals or combinations of signals to assess their responses according to the nature of the signal emitted by the experimenter. We also tested the impact of experience of successive tests. Pigs mainly use auditory signals in their interspecific communication and some visual signals [46]. Based on previous research discussed above, we hypothesize that (1) piglets would be spontaneously able to find a hidden reward in an object-choice task after the use of visual or auditory signals by an experimenter and that combining visual and auditory signals could lead to equivalent or better performances than individual signals, (2) experience could lead piglets to improve their success over subsequent trials of the same object-choice task or between tasks with different signals, and (3) different behavioural responses such as visual and physical interest towards the experimenter and trajectories of walking before visiting the bowls could be observed according to signal type, outcome of the trials (success or failure) and previous experience of the piglets.

## Material and Methods

### Ethical approval

All applicable European, French, and institutional guidelines for the care of animals were followed. All procedures performed in studies involving animals were in accordance with the ethical standards of the French National Institute for Agricultural Research and received an approval from the regional ethic committee (C2EA– 07 Comité rennais d’éthique en matière d’expérimentation animale).

### Subjects and housing

Thirty female piglets, Pietrain x (Large White x Landrace), from the experimental herd of the INRA joint research unit 1348 PEGASE (Saint-Gilles, France) were involved in the experiment. They were chosen according to mean weaning weight and dispersion of their batch (batch (n = 66): M ± S.E.M = 8.5 ± 1.9 kg; sample (n = 30): M ± S.E.M = 8.3 ± 2.0 kg), and had no visible health problems. Piglets were weaned at four weeks of age and transferred to their experimental pens. Weaned piglets were housed in groups of five: two from one litter, and three from another. The fully slatted pens (1.2 x 1.3 m) were equipped with chains as enrichment material, a feeder (0.6 x 0.2 m) and a drinking bowl. Water and commercial pelleted food were provided *ad libitum* except during testing. Ambient temperature was on average 25.5°C, decreasing from 28°C to 23°C over the post-weaning period. Artificial light was provided from 8 a.m. to 5 p.m. During the experiment, two piglets had to be excluded due to a strong stress reaction to social isolation and diarrhoea. In total, 28 piglets were submitted to the different phases of the experiment.

### General procedure

**Fig 1:**
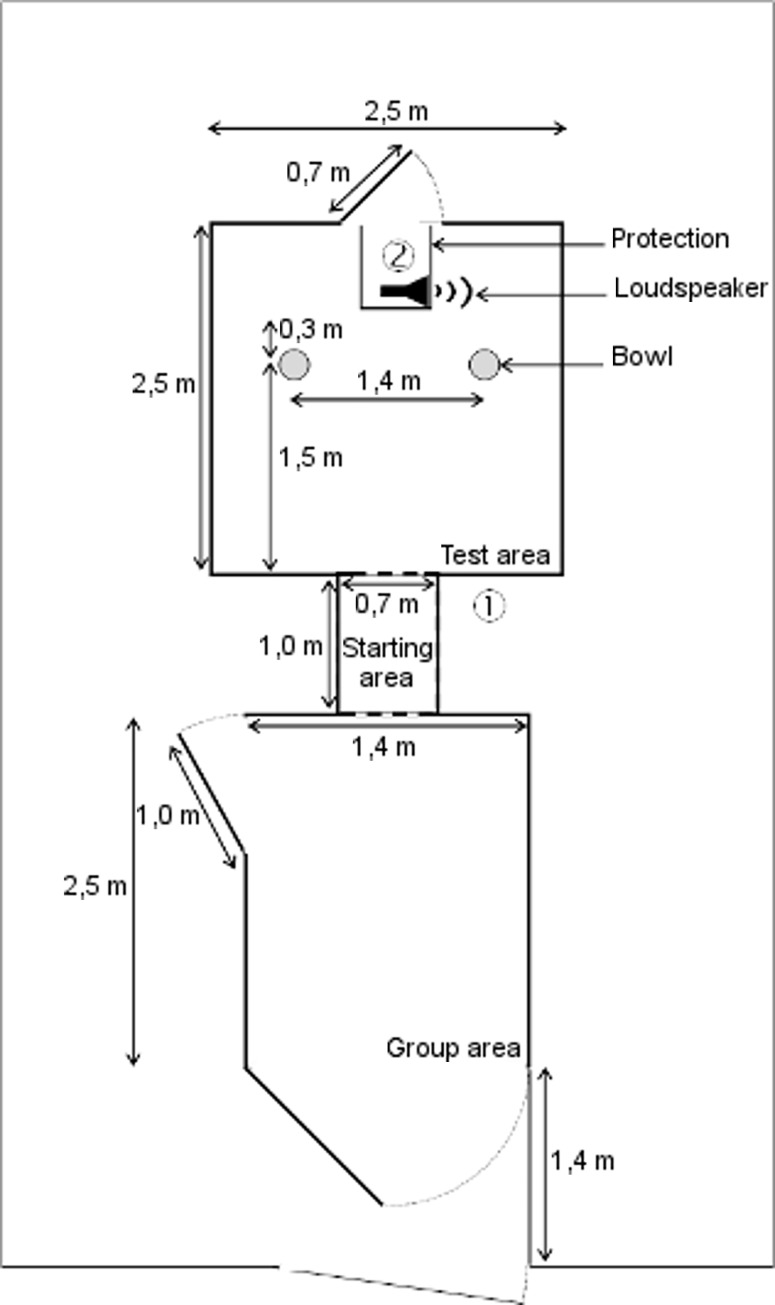
Dimensions and equipment of the test arena. Circled numbers indicate experimenters; the suspended camera above the test area door is not represented

Training and tests were conducted in a room adjacent to the home pen room. The test arena consisted of opaque plastic walls and a concrete floor (Fig 1) divided into three areas separated by sliding doors opened by experimenter 1. The three areas were a group area at the entrance to hold the littermates of the test piglet, a starting area where the test piglet was held immediately before the test, and a test area. The group area was covered with sawdust and enriched with two toys made of plastic and strings hanging on the walls.

**Fig 2:**
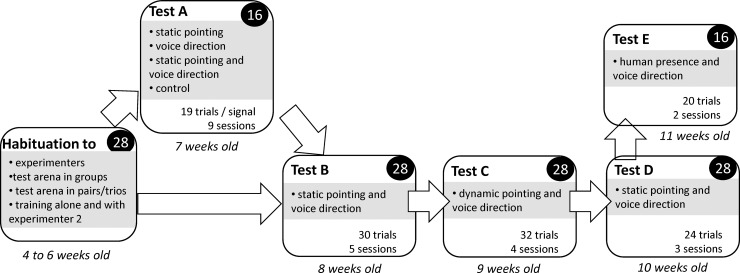
The general procedure was divided into five test steps: description of their principle. Numbers of animals involved are written in black circles and types of signals broadcast are specified inside test steps.

The general procedure of the experiment was adapted from Nawroth et al. [42] (Fig 2). Piglets were familiarised over a period of two weeks to reduce their fear of the experimenters and the test area. Piglets were trained to visit the bowls in the test area while alone and with the experimenter. Sixteen piglets were then selected and submitted to their first test, the “previous signals exposure test” (test A), from their seventh week of age. At the end of this test, the 28 piglets were submitted to three successive tests: the first presentation of a static combination of signals test; the dynamic combination of signals test; and the second presentation of a static combination of signals test. 16 piglets were then selected and submitted to the voice direction test.

### Familiarisation and training period to the experimental procedure

Animals became accustomed to both experimenters over four days (day 3 to 7): each experimenter spent 11 min/ day in each pen: 30 s standing, 30 s sitting on a bucket and then giving piglets hand contact for two minutes per piglet. Contact was given using gradual steps: reaching out, touching the piglet’s body with finger tips and then full hand, caressing the body and finally scratching the body. If piglets interrupted a step by moving away, the experimenter started again from the first step. Animals stepping on the experimenters or hurting them (biting, pushing) were gently pushed away. It was also necessary to familiarise animals to female auditory signals to avoid any disturbance due to novelty on the first test sessions. We therefore broadcasted two playbacks: one of the female voice used in the tests and one from another female voice (test voice: 248 ± 30 Hz; non-test voice 2: 204 ± 32 Hz). Playbacks consisted of three recordings of the two voices: the test procedure sentence pronounced in a neutral tone, and tongue clicking and whistling in the same rhythm as the sentence. Playbacks were broadcasted, alternating the two voices in the pens, for two hours on the day before the first test and for one hour on the first test day, before the first test. The same loudspeaker in the test arena was used throughout.

During days 11 to 13, we habituated the piglets to being moved as a group to the testing arena. Once per day, all piglets from the same pen were moved together towards the group area (Table 1). Plastic boards were used by experimenter 1 to guide the piglets to the group area. Thereafter each group was allowed to visit the whole testing arena for 15 min for two sessions. Two bowls (Ø 0.20 m) separated by 1.40 m were fixed to the floor (experiment 4 in [42]). They were placed 1.50 m from the entrance of the starting area (Fig 1). The bowls contained a removable dark plastic bottom to prevent piglets from seeing the reward position. One of the bowls contained five rewards (M&M’s®); with the rewarding side being balanced across sessions. In the following days (12 and 13), groups were divided into pairs and trios to habituate animals to being in smaller groups. Pairs and trios explored the starting and test areas for 10 min, while the other group of piglets remained in the group area (Table 1). One of the bowls contained two or three rewards per pair or trio respectively; the side containing the rewards was balanced across sessions. Our aim was to increase their interest in visiting the bowls either through their own experience of being rewarded for visiting the bowls, or through their observation of other piglets being rewarded for doing so. Getting individual access to a reward was assured in the following training period.

**Table 1:** Timeline of the familiarisation period: familiarisation steps from weaning day (D0) with their duration and frequency.

Day	Step	Duration and frequency
0	Weaning and transfer to home pen	1 h
3 to 7	Familiarisation to experimenter	2 min/ piglet + 1 min for the group/ day = 11 min for the group/ day
8	Familiarisation to experimenter	2 min/ piglet + 1 min for the group = 11 min for the group
	Group moving to test arena and group familiarisation to the test arena	1 x 15 min/ group
9–10	Resting days	2 days
11	Group moving to group area and group familiarisation to the test arena	2 x 15 min/ group
12–13	Group moving to group area and pairs/ trio familiarisation to the test arena and the starting area	10 min/ group/ day
14	Group moving to group area and individual familiarisation to the testing situation	8 sessions alone/ piglet + 2 sessions with experimenter 2
	Familiarisation with female auditory signals	2 h
15	Familiarisation with female auditory signals	1 h
	Start of “previous signals exposure test”	

**Fig 3:**
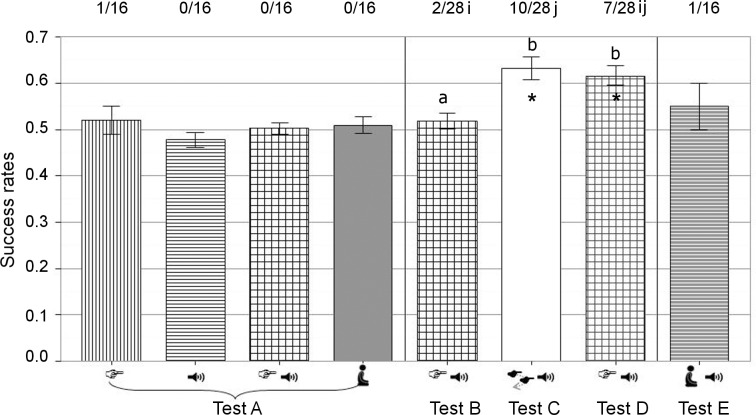
Correct choices according to the signal given by the experimenter in each test. Bars represent mean success rates (± S.E.M.) for tests A, B, C and D, or median success rate (Q25-Q75) for test E. *: p < 0.05 correspond to values above chance level at group level. Bars with different letters are significantly different (*p* < 0.05). Numbers above bars indicate the numbers of individuals that reached the success criterion over the total number of subjects that participated and numbers with different letters are significantly different (*p* < 0.05). Types of signals: S. Pointing: static pointing gesture, Voice: voice directed to the reward, S. pointing & Voice: static pointing gesture and voice directed to the reward directed to the reward; D. pointing & Voice: dynamic pointing gesture and voice directed to the reward, Voice & Presence: voice directed to the reward and experimenter’s presence. Test A: “previous signals exposure test”, test B: pointing and voice test 1, test C: dynamic pointing and voice test, test D: pointing and voice test 2, test E: voice test.

On day 14, we proceeded to an individual familiarisation to the testing situations. Each piglet from a group was individually introduced into the starting area for a session of 10 consecutive training trials. During the first eight trials the piglet was alone in the test area with one reward bowl—experimenter 2 was hidden from sight outside the test area. In the last two training trials experimenter 2 was present in the test area, kneeling motionless and looking straight forward. The reward side was balanced across trials. A bell attached to the test area door was used by experimenter 1 to attract the piglet’s attention and to encourage the piglet to move towards the test area. Piglets had maximum of 90 s to visit both bowls, i.e. put its snout in the bowls. The piglet was then gently pushed to the starting area. During the first eight trials (piglet alone), if an animal did not visit both bowls after 90 s over two consecutive trials, it was trained as in the two last trials (in the presence of experimenter 2). If during these trials it did not visit both bowls again, it was eliminated from the selection. This happened for nine piglets. If the piglet visited both bowls, it was submitted to remaining training trials (i.e. alone). Three other animals were excluded because they chose the same side first while entering the test area for each training trial (*p =* 0.008, binomial bilateral test). Finally 16 piglets were used in the “previous signals exposure test” (Fig 3) and were considered as belonging to the “signals+” piglet group, while the remaining 12 piglets were not submitted to the “previous signals exposure test”, defined as the “signals-”group.

### Test conditions

After the familiarisation period, all tests took place using the same procedure: each group of tested piglets was moved from their home pen to the group area. The piglet to be tested was then introduced into the starting area by experimenter 1. A protective plastic board (0.4 m high with two metal gridded sides to ensure sound broadcasting) was present in the test area to protect the loudspeaker and prevent piglets from climbing on experimenter 2 who gave pointing signals. It allowed piglets to see above the hips of experimenter 2. A trial was ended either by a visit to a bowl–where the piglet touched a bowl or the 0.05 m wide disc around the bowl with its snout—or after 90 s if the piglet did not visit a bowl. Piglets which did not visit a bowl were pushed back to the group area by experimenter 2, and the rest of their training session was completed the following day. When a trial ended with a visit, the piglets could eat the reward if they visited the reward bowl first and were then pushed by experimenter 2 to the starting area to start a new trial or to the group area at the end of a test session. Experimenter 2 cleaned the bowls to prevent saliva or pheromone traces from misguiding piglets. She then put the reward in the bowl according to experimenter 1’s visual indications (pointing to the bowls). The opaque starting areas walls kept piglets from seeing this preparation phase. The rewarding sides were balanced throughout with rewards placed on the same side no more than twice consecutively in any one session. Our objective was to submit piglets to at least 20 trials per signal as in the tests in Nawroth et al. [42]. We adapted the numbers of trials per daily session between tests according to piglets’ signs of stress assessed by the occurrence of high-pitched screams and escape behaviour. The total number of trials was identical, however, trials per day varied slightly as sessions were postponed until the following day for piglets showing signs of stress.

#### “Previous signals exposure test” (test A)

The test consisted of alternative exposure to four different signals:

- “Static pointing gesture”: experimenter 2 kneeled behind the protective board facing the piglet and pointed her finger toward the rewarding bowl (ipsilateral arm) before the piglet entered. The tip of her finger was situated about 0.3 m away from the bowl. She looked at a point above the starting area door to avoid eye contact. No sound was broadcasted.
- “Voice directed to the reward”: experimenter 2 placed the loudspeaker behind the protective board directed to the rewarding side and left the test area. Sound was broadcasted from 3 s before the entrance of the piglet until the end of the trial. The broadcast consisted of a female voice (315 ± 47 Hz) speaking for 3.35 s. The sentence was pronounced on an encouraging tone: “De ce côté, cochon. Allez. Par ici. Viens là” (UK version “On this side, pig. Come on. Over here. Come here”. Animals were accustomed mainly to male voices in the facilities therefore we chose an average female voice to minimise the influence of a previous voice experience. The voice was recorded with a microphone (MKH 50 P 48 Sennheiser, Germany) with a microphone module and an acoustic foam windscreen (K6/ME66 Sennheiser, Germany). The microphone was connected to an audio digital portable recorder (Portable Solid State recorder, PMD661 Marantz Professional, The Netherlands; amplitude resolution: 16 bits; sampling rate: 44.1 kHz) in WAV format. Recordings were prepared and fundamental frequency analysed with the Praat software (5.2.01, www.praat.org). Playbacks were broadcasted using a loudspeaker (MA-100su Mipro Electronics, Taiwan) at 80 ± 2 dB max sound pressure level (at 1 m from the source, determined using a sound level meter, SL-100 Voltcraft, Germany). At the end of a trial experimenter 2 (who stood outside during the trial) entered the test area when signalled by experimenter 1 to stop the broadcast and push back the piglet.
- “Static pointing gesture and voice directed towards the reward”: experimenter 2 kneeled behind the protective board and placed the loudspeaker in front of her knees, behind the protection and directed it to the rewarding side. Sound was broadcasted from 3 s before the entrance of the piglet. The kneeling experimenter pointed her finger toward the rewarding bowl (ipsilateral arm) before the piglet entered. She was facing the piglet and fixed her gaze on a point above the starting area door to avoid eye contact. The tip of the experimenter’s finger was situated at about 0.3 m of the bowl. The voice signal was similar to the preceding signal.
- “Control”: experimenter 2 kneeled motionless behind the protective board, her arms resting on her thighs and she fixed her gaze on a point above the starting area door. No sound was broadcasted.

Sixteen piglets were tested in the “previous signals exposure test” (test A). The test lasted for 9 sessions distributed over 7 days. Piglets were submitted to at least one session per day with two additional sessions distributed over two days (balanced across pen groups and days of testing). We started with three trials per signal (12 trials per session) on the first day but piglets showed signs of stress at the end of the session. We consequently adjusted the number of trials for the subsequent days and kept it to 10 trials per session. Each session included two training trials without the experimenter followed by two trials per signal displayed in a balanced order with no more than two identical consecutive signals in the same test session. In total, piglets received 19 trials per signal.

#### Tests with combinations of voice direction and pointing gesture, either static (tests B and D) or dynamic (test C)

Tests B (pointing and voice test 1), C (dynamic pointing and voice test) and D (pointing and voice test 2) involved all 28 piglets. Each test consisted of only one type of combined signal: voice and static pointing gesture (test B and D), or voice and dynamic pointing gesture (test C). The aims were to determine the effect of simplifying the testing conditions (one type of signal only), to test the use of combinations of signals, and to test the effect of test experience on the results.

In the first “pointing and voice” test (test B) piglets were submitted to the “static pointing gesture and voice directed to the reward” signal, presented in the same conditions as in the “previous signals exposure test” (test A). None of the 16 piglets involved in test A had achieved the success criterion (i.e. had performed more successful trials than expected by chance, see data analysis) after 19 trials, thus we chose to reach a number of 30 trials which is the maximum number of training sessions piglets were submitted to in Nawroth et al. [42]. The test therefore lasted for five days with one session of six trials per day (30 trials in total). For this test the bowls were covered with a grey plastic pipe (0.3 m high, Ø 0.28 m) to prevent piglets from accessing the reward by themselves. Experimenter 2 removed the first visited pipe to show the bowl to the piglet and let it eat the reward if present. The piglet was then pushed back to the starting area by experimenter 2. She then cleaned the bowls and the pipes before rewarding one bowl again for the next trial according to a randomised order.

In the dynamic pointing and voice test (test C) piglets were submitted to a “dynamic pointing gesture and voice directed to the reward” signal. The test lasted for four days with one session of eight trials per day (32 trials in total). The signal was similar to the static pointing gesture with voice except that when the door opened, experimenter 2 repeatedly and slowly moved her arm to the rewarding pipe until the piglet had made a choice. The experimenter then dropped the reward in the pipe, stopped the broadcast, and removed the pipe to show to the piglet its choice. Experimenter 2 then pushed back the piglet to the starting area, cleaned the bowls and pipes and rewarded one of them on experimenter 1’s indications.

In the second “pointing and voice” test (test D) piglets were submitted to the “static pointing gesture and voice directed to the reward” similarly to test B. The test lasted for three days with one session of eight trials per day (24 trials in total). From the results of test C we considered 24 trials to be sufficient to calculate a success rate.

#### Voice direction only test (test E)

Test E (voice test) was the last test. Only voice direction was used to indicate reward location to determine if this signal could be sufficient for piglets, in presence of experimenter 2. Sound was broadcasted from 3 s before the entrance of the piglet until the end of the trial. The experimenter kneeled motionless with her arms resting on her thighs and she fixed her gaze on a point above the starting area door. Sixteen piglets were submitted to the test which lasted for two days with one session of 10 trials per day (20 trials in total). We had only two days left before transfer to the finishing pens, and thus could not test all the piglets. The sixteen piglets were selected due to their absence of side bias in the pointing and voice test 2and their results in the dynamic pointing and voice test: 8 fulfilled the success criterion (21/32 successful trials) which provided a balanced group.

### Behavioural recordings

Table 2 summarises the behavioural recordings. Experimenter 1 recorded the latency to visit the first bowl and the side of the visit (Table 2), experimenter 2 recorded the looks at experimenter 2 and the physical contacts with her. These behavioural parameters were scored live. The trajectories to the bowl from the exit of the starting area were determined retrospectively from video recordings for the “previous signals exposure test”, and scored live by experimenter 1 for tests B to D.

**Table 2:** Ethogram of the observed behaviours during tests.

Criteria	Description
**Animal’s first visit side**	*Left*: animal had its snout within a max distance of 0.5 m around the left bowl
	*Right*: animal had its snout within a max distance of 0.5 m around the right bowl
**Latency to visit the first bowl**	Latency (seconds) from the opening of the starting area door until animal made a first visit
**Look at the experimenter**	*Absent*: no look at all after entry
	*Glance*: look less than 1 s after entry
	*Stare*: look more than 1s after entry
**Contact with the experimenter**	*Presence*: Any form of direct contact with experimenter 2: sniffing, touching, biting, stepping…
	*Absence*: no contact with experimenter 2
**Trajectory**	*Direct* trajectory: straight walk to the bowl
	*Indirect*: non-straight walk to the bowl. Any contact with experimenter 2 before a visit was considered as an indirect trajectory.

### Response evaluation

If the animal’s first visit to a bowl was the rewarded one, the trial was defined as successful, otherwise as failed. The individual success rate was defined as the ratio of successful trials to the total number of trials. This rate was calculated for each signal in the “previous signals exposure test”, each session, and each test in the other tests. The proportions of looks, glances and stares at the experimenter before visiting a bowl were determined for each individual and per test for successful and failed trials, and per session. They were determined as the ratio of the number of looks, glances or stares to the total number of trials. The number of contacts with the experimenter and the total proportion of direct and indirect trajectories before visiting the first bowl were determined for each piglet by signal (test A) or test (test B to E), for successful and failed trials.

### Data analysis

The R software [47] was used for the statistical analysis of data.

#### The “previous signals exposure test” (test A)

Individual success rates were considered as significantly deviating from chance level for each signal when the number of successful trials was strictly above 13/19 trials (one-tailed binomial test, *p* = 0.03). Mixed linear models were used to analyse fixed factor effects and their interactions on the success rates and on the individual latencies to visit the first bowl. “Signal” was used as a fixed factor and “piglet” as random effect in the nlme package [48]. As conditions for parametric analyses were not met, even after transformation of the data, we used non parametric methods–Friedman and Mann and Whitney tests—to compare the individual latencies to visit the first bowl between signals for each trial outcome. We ran the same analysis for the individual proportions of total looks and stares at the experimenter between trials for each signal with a Mann and Whitney test and then between the types of signals with a Friedman test. In both cases, we excluded the voice directed to the reward signals. Indeed in this case experimenter 2 was outside the test area. We ran the same analysis for the individual proportions of direct trajectories for each signal. Individual side preference was considered for each signal when the number of first visits to a bowl on the same side was strictly above 14/19 trials (two-tailed binomial test, *p* = 0.02).

#### Voice directed to the reward and variation of the quality of the pointing gesture: test B, C and D

At the group level, we compared the distributions of piglets achieving the success criterion or not between the test B, test C and test D with a *χ*^2^ test and Fisher tests for two by two comparisons. We ran the same analyses for the distributions of total number of glances and stares at the experimenter between the three tests. As data were too scarce to perform statistical analysis, we can only report for each test the proportions of piglets reaching the success criterion and making/not making contact with the experimenter in at least one trial. Taking into account the outcome of the trial, a comparison between the number of animals which never used indirect trajectories and those which used at least one indirect trajectory in each test was made using Fisher’s exact tests.

On an individual level, a side preference was considered to be present when the number of first visits to a bowl on the same side was significantly higher than it would be by chance (two-tailed binomial test, test B: 21/30 trials, *p* = 0.04; test C: 23/32 trials, *p* = 0.02; test D: 18/24 trials, *p* = 0.02). We compared the side preference distributions of animals between signals+ and signals- piglets with Fisher’s exact test and between tests with a *χ*^2^ test. Individual number of successful trials was considered as significantly different from chance level when above a determined threshold (one-tailed binomial test, test B: 19/30 trials, *p* = 0.05; test C: 21/32 trials, *p* = 0.03; test D: 16/24 trials, *p* = 0.03). We analysed the factors’ effects and their interactions with mixed linear models for the individual success rates and the individual latencies to visit the first bowl. We found no significant interaction between piglet’s group (signals+ and signals-) and trial outcome, thus it was removed from all the models. We ran the same analysis on the individual proportions of looks and stares at the experimenter transformed using the arcsine function to meet the ANOVA requirements. We used the nlme package [48] with “piglet” as random effect. Least square means were compared as post-hoc analyses using the lsmeans package [49]. We ran a first set of analyses to compare data between the test B, C and D. We included in the model concerning the individual success rates the following factors as fixed effects: “test”, “previous signals exposition” (signals+ and signals- groups) and their interaction. We added in the models concerning the individual latencies to visit the first bowl a “trial outcome” (successful or failed) factor and its interaction with the “test” factor. We ran the same analysis for the individual proportions of looks and the individual proportions of stares at the experimenter. A second set of analyses compared data between the sessions of each test, repeating the same models for each test with a “session” factor instead of the “test” factor.

#### Voice test (test E)

Individual success rates deviated significantly from chance level when the number of successful trials was strictly above 14/20 trials (one-tailed binomial test, *p* = 0.021). As conditions for parametric analyses were not met, we used Wilcoxon and Mann and Whitney tests to compare the individual success rates between sessions, previous signals exposure, groups of piglets, and trial outcome. We ran the same analysis for the individual latencies to visit the first bowl, the individual proportions of looks or stares, and the proportions of direct trajectories. Individual side preference was significant when the number of first visit to a bowl on the same side was strictly above 15/20 trials (two-tailed binomial test, *p* = 0.04).

## Results

### Spontaneous responses to voice direction, static pointing and their combination (test A, “previous signals exposure test”)

One piglet out of 16 tested reached the success criterion with the static pointing gesture (15/19 successful trials, *p* = 0.01). No piglet reached the success criterion for the other types of signals or for the control situation: all piglets succeeded in less than 13 trials. There were no differences between the types of signals on the individual success rates which were on average at chance level (M ± S.E.M. = 0.50 ± 0.01, *F*_3, 42_ = 0.97; *p* = 0.42; Fig 3).

It took about 3 s for piglets to reach the first bowl (Table 3). Comparisons between trial outcomes revealed that piglets were quicker (-29%) at visiting the empty bowl first than the rewarded bowl with the static pointing gesture (*W* = 24, *p* = 0.04; Table 3). There were no differences between the individual latencies to visit first the rewarded and the empty bowl for the other signals (voice direction: *W* = 36, *p* = 0.19; static pointing gesture and voice: *W* = 65, *p* = 0.45; control: *W* = 58, *p* = 0.93). Piglets took significantly more time to visit the empty bowl with the static pointing gesture than with the combination of static pointing gesture and human voice (+27%, *p* = 0.05). There was no difference for successful trials (*p* = 0.08).

**Table 3:** Results on individual latencies (s) to the first visit of a bowl, main statistical effects and probabilities. Results are presented either by mean ± S.E.M. or median and interquartile (Q25–Q75). Friedman statistics are presented for test A: the “previous signals exposure test”; Fisher’s statistics for test B: the pointing and voice test 1, for test C: the dynamic pointing and voice test, for test D: the pointing and voice test 2, and for the comparison between test B, C and D; Wilcoxon and Mann and Whitney statistics for test E: the voice direction test. Values with different bold letters are significantly different on a same row and between two rows for groups of piglets.

Test, (number of animals)	Factor ^a^	Type of signal ^b^ and session (S)					Factors, statistics and probabilities for all tested effects				
							Test or session	Test A±	Trial outcome	Test or session x test A±	Test x trial outcome
**A (n = 15)**	Trial outcome:	S.P. S1 to S9	V. S1 to S9	S.P. & V. S1 to S9	Control S1 to S9						
	Success	3.4 (2.6 - 3.8)	3.3 (2.8 - 4.0)	3.2 (2.4 - 3.6)	3.3 (2.9 - 4.9)				6.84		
	Failing	4.2 **a** (3.3 - 5.6)	3.0 **abc** (2.6 - 3.7)	3.0 **c** (2.6 - 3.8)	3.4 **ab** (3.0 - 4.2)				8.04*		
**B, C and D (n = 28)**	Group:	B: S.P. & V. S1 to S5	C: D.P. & V. S1 to S4	D: S.P. & V. S1 to S3			20.84***	2.51	3.33	8.11***	1.61
	Signals+	3.4 ± 0.3 **a**	2.5 ± 0.3 **a**	3.26 ± 0.3 **a**							
	Signals-	5.3 ± 0.4 **b**	2.8 ± 0.4 **a**	3.1 ± 0.4 **a**							
**B (n = 28)**	Session:	S.P. & V. S1	S.P. & V. S2	S.P. & V. S3	S.P. & V. S4	S.P. & V. S5	4.75***	3.91	2.41	1.32	0.52
		5.3 ± 0.7 **ab**	6.2 ± 0.7 **b**	4.9 ± 0.7 **ab**	2.9 ± 0.7 **a**	2.8 ± 0.7 **a**					
**C (n = 28)**	Group:	D.P. & V. S1	D.P. & V. S2	D.P. & V. S3	D.P. & V. S4		9.51***	1.02	0.06	3.66*	0.71
	Signals+	2.8 ± 0.2 **abc**	2.4 ± 0.2 **abc**	2.4 ± 0.2 **abc**	2.3 ± 0.2 **abc**						
	Signals-	3.5 ± 0.3 **c**	3.1 ± 0.3 **bc**	2.2 ± 0.3 **a**	2.2 ± 0.3 **ab**						
**D (n = 28)**	Session:	S.P. & V. S1	S.P. & V. S2	S.P. & V. S3			1.24	0.05	2.10	0.30	0.03
		2.8 ± 0.2	3.4 ± 0.4	3.4 ± 0.3							
**E (n = 16)**	Session:	V. & Pr. S1	V. & Pr. S2				39	34	302		
		3.0 (2.4 - 3.5)	3.3 (2.6 - 4.1)								

^a^ Factors: Success: successful trial outcome, Group: Signals+ piglets: piglets involved in the “previous signals exposure test”, Signals- piglets: piglets not involved in the “previous signals exposure test”.

^b^ Type of signals: S.P.: static pointing gesture, V.: voice directed to the reward, S.P. & V.: static pointing gesture and voice directed to the reward; D.P. & V.: dynamic pointing gesture and voice directed to the reward; V. & Pr.: voice directed to the reward and experimenter’s presence.

*: *p* < 0.05

**: *p* < 0.01

***: *p* < 0.001.

Piglets looked at the experimenter in almost all trials and for all tested types of signals (min: 91 to max: 94% of trials per signal). Piglets looked in the same proportions, for successful and failed trials, for each type of signal (static pointing gesture: *W* = 8.5, *p* = 0.75; static pointing gesture and human voice: *W* = 21, *p* = 0.27; control: *W* = 15, *p* = 0.93). There was no influence of the type of signal on the proportion of looks (Md = 1.00 (0.95–1.00); Friedman *F* = 1.03, df = 2 *p* = 0.60). The trial outcome did not influence the proportion of stares for each type of signal (static pointing gesture: *W* = 52, *p* = 0.32; static pointing gesture and human voice: *W* = 26, *p* = 0.92; control: *W* = 56, *p* = 0.85), or between types of signal (Md = 0.42 (0.37–0.53); Friedman *F* = 2.06; df = 2; *p* = 0.36). Contacts with the experimenter before choosing a bowl were scarce; four piglets made contact with the experimenter eight times while she was providing the static pointing gesture, one piglet contacted the experimenter once while she was providing the static pointing gesture and human voice and another piglet did so once during the control signal.

Trajectories before visiting a bowl were mostly direct (min: 57 to max: 67% of trials per signal). We found no association between the trial outcome and the proportions of direct trajectories (static pointing gesture: *W* = 73, *p* = 0.21; voice directed to the reward: *W* = 59, *p* = 0.36; static pointing gesture and human voice: *W* = 58, *p* = 0.92; control: *W* = 28, *p* = 0.69). The type of signal influenced the proportion of direct trajectories (Friedman *F =* 9.15; df = 3; *p* = 0.03) but pairwise comparisons revealed these all to be non-significant (Md = 0.62 (0.44–0.86); p > 0.05).

For each signal four out of 16 piglets had no side preference, among them three piglets did not present a side preference for any signal. The majority of piglets went to the left bowl first.

### Responses to the multi-signal tests: pointing and voice test 1, dynamic pointing and voice test, pointing and voice test 2 (tests B, C, D)

On 28 tested piglets, one piglet achieved the success criterion for the three tests, two piglets achieved the success criterion for two tests, and 12 piglets succeeded in one test. One piglet chose the empty bowl over the rewarded one, this was during test B. All other animals performed at chance level in all tests. The proportion of piglets reaching the success criterion significantly increased between test B (2/28) and C (10/28; *p* = 0.02); the number of piglets succeeding in test D (7/28) was in-between (Fig 3, *χ^2^* = 6.67; df = 2; *p* = 0.04, Fig 3). The individual success rates were higher for test C and D than for test B (*F*_2, 52_ = 10.56, *p* < 0.01; Fig 3). Animals did not succeed as a group for any test as the number of animals succeeding was always under 18/28 (*p* > 0.96). However, the total number of successful trials was higher than statistically expected for test C and D (test B: 433/836 *p* = 0.16; test C: 566/896 *p* < 0.01; test D: 413/671 *p* < 0.01). Latencies to visit the first bowl were higher for test B than for the subsequent tests (*p* < 0.01, Table 3). There was no effect of the trial outcome on the individual latencies to first visit neither in the statistical model including tests B to D (*p* = 0.07; successful trials M = 3.40 s ± 0.14 s; failed trials M = 3.23 s ± 0.13 s) nor in models done by test (test B: *p* = 0.12, test C: *p* = 0.80, test D: *p* = 0.15). There was no interaction between test and trial outcome (*p* = 0.20).

**Fig 4:**
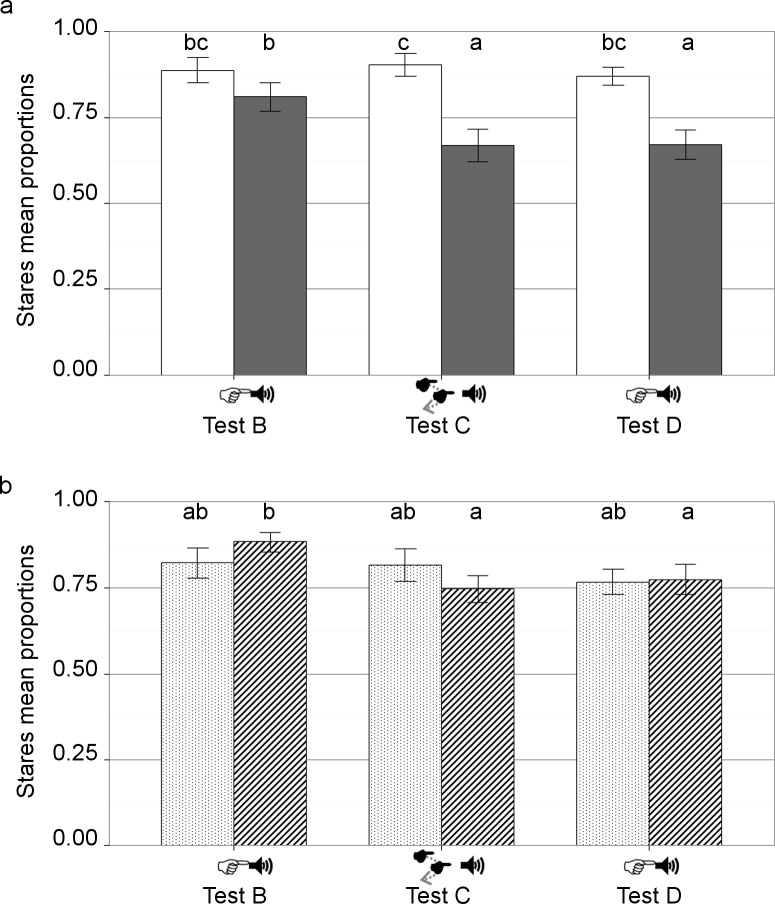
Stares at the experimenter differed according to trial outcomes and between piglet groups (n = 28). (a) Mean proportions of stares (± S.E.M.) are presented according to trial outcomes. White bars represent successful trials, grey bars represent failed trials. (b) Mean proportions of stares (± S.E.M.) are presented according to previous signal exposure. Dotted bars represent signals+ piglets previously exposed to signals in test A, striped bars represent signals- piglets not exposed to test A. Bars with different letters are significantly different (*p* < 0.05). Types of signals: S. pointing & Voice: static pointing gesture and voice directed to the reward; D. pointing & Voice: dynamic pointing gesture and voice directed to the reward. Tests: test B: pointing and voice test 1, test C: dynamic pointing and voice test, test D: pointing and voice test 2.

Piglets looked at the experimenter in almost all trials (min: 93% to max 94% of trials in first presentation of a static combination of signals test, the dynamic combination of signals test and the second presentation of a static combination of signals test). There was no significant effect of the test (F_2, 133_ = 0.65, p = 0.52) on the proportion of looks. The individual proportions of looks at the experimenter were significantly higher (+6.7%) in successful trials than in failed trials (successful trials M = 96 ± 1%; failed trials M = 90 ± 2%, F_1, 133_ = 33.29, p < 0.01; Fig 4A). If we consider only the trials with looks at the experimenter, glance and stare proportions significantly differed according to the test (*χ^2^* = 24.38; df = 2; *p* < 0.001): stares in test B (92%) significantly decreased by 5,4% in test C (87%, *χ^2^* = 12.61; df = 1; *p* = < 0.001) and by 8.7% in test D (84%, *χ^2^* = 23.11; df = 1; *p* = < 0.001), stares in test C and D did not differ significantly (*χ^2^* = 2.06; df = 1; *p* = 0.15). The individual proportions of stares during failed trials were significantly higher during test B than during the other tests (interaction between test and trial outcome: *F*_2, 133_ = 5.43, *p* < 0.01) and these proportions were also higher in successful trials than in failed trials for test C and D, but not for test B. We also found a significant effect of the test (*F*_2, 133_ = 8.75, *p* < 0.01) and of trial outcome (*F*_1, 133_ = 86.82, *p* < 0.01) on the individual proportions of stares at the experimenter.

There is no evidence of a clear link between contact with the experimenter and reaching the test success criterion. However, for test B, one in every two piglets who reached the success criterion made contact with the experimenter at least once before choosing a bowl *versus* 11/26 piglets who did not reach the success criterion. In test C, this represented two piglets out of six making contact with the experimenter *versus* 22/22 and in test D, six piglets out of seven making contact with the experimenter *versus* seven out of 22.

Indirect trajectories before the first visit to a bowl were scarce (min: 4 to max: 9% of trials per test) but piglets did not perform direct and indirect trajectories equally in all three tests (*χ^2^* = 16.93; df = 2; *p* < 0.001). Direct trajectories in test D (91% of trials) were 5.2% lower than in test B (96%, *χ^2^* = 13.37; df = 1; *p* < 0.001) and 4.2% lower than in test C (95%, *χ^2^* = 8.83; df = 1; *p* < 0.01). There was no significant difference between tests B and C (*χ^2^* = 0.49; df = 1; *p* = 0.48). We did not find any association between type of looks and trajectories for any test (test B: 92% of direct trajectories were associated with stares and 94% of indirect trajectories, *p* = 1; test C: 87% of direct trajectories were associated with stares and 89% of indirect trajectories, *p* = 0.82; test D: 83% of direct trajectories were associated with stares and 88% of indirect trajectories, *p* = 0.46). Piglets performing indirect trajectories had a two to three times higher chance of success in a trial: indirect trajectories in a trial were significantly more closely associated with successful trials (test B: *χ^2^* = 5.46; df = 1; *p* = 0.02; successful trials: 6% of indirect trajectories, failed trials: 3%, test C: *χ^2^* = 5.46; df = 1; *p* = 0.02 successful trials: 7%, failed trials: 3%, test D: *χ^2^* = 10.9; df = 1; *p* < 0.01; successful trials: 12%, failed trials: 4%). However, one of the two animals that passed the first presentation of test B only performed direct trajectories; 13 piglets that did not pass the test performed at least one indirect trajectory. We found no association between the use of at least one indirect trajectory and achieving the success criterion, either for test C (*p* = 1; 50% of piglets performed at least one indirect trajectory) or for test D (*p* = 0.06; 68% of piglets performed at least one indirect trajectory).

On 28 tested piglets, eight piglets did not show a side preference, eight piglets showed a side preference in one test, six piglets in two tests and six piglets in three tests. Eleven piglets did not show a side preference in test B, 12 in test C and 15 in test D. The majority of piglets went to the left bowl first in tests B and C and to the right bowl first in test D.

### Responses to human voice provided alone in human presence (test E)

Only one piglet out of 16 reached the success criterion in test E (15/20 successful trials, *p* = 0.02), the other piglets succeeded in fewer than 12 trials. We did not observe any contact with the experimenter during the voice direction test. The individual proportions of direct trajectories did not significantly differ according to trial outcome (*W* = 12, *p* = 0.83, Md = 100% (89%–100%)). The individual latencies to visit the first bowl were not statistically influenced by the trial outcome (*V* = 301.5, *p* = 0.16, Md = 3.00 (2.20–3.80)). Trial outcome did not affect the individual proportions of looks at the experimenter (*V* = 9.5, *p* = 0.92). In 16 tested piglets, thirteen piglets did not show a side preference, one piglet went to the left bowl first and two on the right.

### Evolution of responses across sessions of a same test (tests B, C, D, E)

We found no effect of session on success rate (test B: *F*_4, 104_ = 0.27, *p* = 0.90; test C: *F*_3, 78_ = 1.48, *p* = 0.23; test D: *F*_2, 52_ = 0.25, *p* = 0.78; test E: *V* = 37, *p* = 0.91, Md = 0.50 (0.50–0.67). For test B, the latencies to visit the first bowl were significantly twice as long for the second session as for the two last sessions (*p* = 0.001, Table 3). The latencies to visit the first bowl in the first and third sessions were intermediate. There was no interaction between session and trial outcome (*p* = 0.72). In test C, there was also a significant effect of session on latencies to first visit (Table 3) but there was no interaction between session and trial outcome (*p* = 0.55). In test D, there was no effect of session (*p* = 0.29) and no interaction between session and trial outcome (*p* = 0.97, Table 3). In test E, The individual latencies to visit the first bowl did not statistically differ between sessions (*p* = 0.24, Table 3). The trial outcomes did not statistically influence the individual latencies to visit the first bowl either during the first session (W = 147, *p* = 0.48) or the second (W = 155.5, *p* = 0.31). We did not find statistical differences between sessions on the individual latencies to visit the first bowl either for successful trials (V = 36, *p* = 0.10) or for failed trials (V = 46, *p* = 0.71).

### Consequences of previous exposure to signals (test A, groups signals+ and signals-) on responses to combinations of signals (tests B, C, D)

Previous signal exposure did not have a significant effect on the individual success rate (test B: F_1, 26_ = 0.12, *p* = 0.73; test C: F_1, 26_ = 0.46, p = 0.50; test D: F_1, 26_ < 0.003, p = 0.96). We found no significant effect on success rate of the interaction between the test factor and the previous signals exposure factor (signals+ versus signals-; *F*_2, 52_ = 0.18, *p* = 0.84).

There was no effect of previous signal exposure on the individual latencies to first visit calculated overall (*p* = 0.12) or by test (test B: *p* = 0.06, test C: p = 0.32, test D: *p* = 0.83). The individual latencies to visit the first bowl were nearly twice as long for test B as for the other two tests in signals- piglets only (interaction between test and previous signal exposure, *p* < 0.01, Table 3).

We found no significant effect of “previous signal exposure” on the individual proportion of looks at the experimenter (F_1, 26_ = 0.001, *p* = 0.98), no significant interactions between the test and previous signals (F_2, 133_ = 0.62, *p* = 0.54), and no significant interaction between test and trial outcome (F_2, 133_ = 1.65, *p* = 0.20). Signals- piglets stared significantly more at the experimenter during test B than during test C and D; there was no significant difference for signals+ piglets (F_2, 133_ = 3.99, *p* = 0.02; Fig 4B). We did not find any effect of previous signals exposure (F_1, 26_ = 0.04, *p* = 0.84) on the proportion of stares at the experimenter.

There was no influence of involvement in test A on side preference for each test (Fisher exact tests: *p* > 0.30) and no difference between tests (*χ^2^* = 1.25; df = 2; *p* = 0.54).

### Consequences of previous exposure to signals (test A, groups signals+ and signals-) on responses to human voice alone (test E)

In test E, previous signal exposure (*W* = 141.5, *p* = 0.56) did not influence the individual success rates which were at chance level (Md = 0.50 (0.50–0.67)). Signals+ piglets did not perform (success rate) better than signals- piglets either during the first session (W = 39, *p* = 0.45) or the second (W = 33, *p* = 0.91). Performances did not statistically differ between the first and the second session for signals+ (V = 15, *p* = 0.93) or signals- piglets (V = 5.5, *p* = 0.68). Previous signal exposure did not influence the individual latencies to visit the first bowl (*W* = 34.0, *p* = 0.83), the proportions of looks to the experimenter before choosing a bowl (*W* = 145.5, *p* = 0.36, Md = 100% (92%–100%)), or the proportion of stares (*W* = 136, *p* = 0.71, Md = 91% (80%–100%)).

### Interactions between sessions experience and previous exposure to signals (test B, C, D)

For each test, we found no significant interaction on the individual success rates between sessions and previous signal exposure (test B: *F*_4, 104_ = 0.55, *p* = 0.70; test C: *F*_4, 78_ = 0.05, *p* = 0.99; test D: *F*_4, 52_ = 0.66, *p* = 0.52). In test C, for signals- piglets only, the individual latencies to visit the first bowl were significantly longer in the first two sessions than in the third session (first session: +59%; second session: +41%, (interaction between sessions and previous signal exposure: test B: *p* = 0.26, test C: *p* = 0.01, test D: *p* = 0.74)). Their individual latencies were also 59% longer for the first session than for the last session.

## Discussion

Initially, piglets did not find the hidden reward in an object-choice task with a combination of auditory and visual signals. After training, they finally succeeded in the task with a combination of two signal types (auditory and visual signal, the latter being provided either statically or dynamically). However, they were not able to use auditory signals alone to find the reward. Piglets did improve their success rate over successive tests, in response to variations in signal type but maybe also in response to testing conditions (i.e. number of sessions). Piglets benefited from a change in the test conditions between “pointing and voice test 1” and “dynamic pointing and voice test” and possibly also from the repetition of the static combination of signals between the “pointing and voice tests” 1 and 2. Previous signal exposure impacted on the latency to visit the first bowl and the looks at the experimenter in the two “pointing and voice tests” and in the “dynamic pointing and voice test”. Moreover we noted associations of behaviour according to trial outcomes (finding the reward or not).

Spontaneously, piglets did not use voice direction, pointing, or a combination of pointing and voice direction to find a hidden reward in our conditions. The latency to visit the first bowl was low in the “previous signals exposure test” (mean 3 to 4.2 s) which might illustrate that piglets understood they had to find the reward in one bowl. Animals were attentive to the experimenter’s presence as they looked at her before choosing in at least 91% of trials but they did not seem to take into account the signals, as the success rates and the number of animals reaching our success criteria remained low. The alternation of visual, auditory or combination of signals in the test may have been troubling for animals, randomly calling to their different senses.

However, piglets reacted differently to the various types of signals emitted during the “previous signals exposure test”, which indicated that they were able to discriminate them, as previously reported in studies on handler discrimination relying on different sensorial cues or signals [34]. The higher level of differences concerned the latency to choose a bowl when the animals failed to find the reward: it was higher with the static pointing gesture than with the other signals. This suggests this signal, associated with an out-stretched arm, might be threatening for piglets or related to an avoidance response to handlers [50]. Nevertheless, the latency was not higher with the combination of static pointing gesture and voice directed to the reward and piglets did not significantly fail more with the static pointing gesture. Thus they did not avoid this signal. Probably that recording eyes orientations or behavioural signs of attention to the experimenter or the signal [21] would help to complete the information.

Despite differences in experimental set-up, our results (no use of a pointing gesture) are in agreement with Albiach-Serrano et al. [41]. On the contrary, pigs used a pointing gesture directed toward a hidden reward in Nawroth et al. [42]. Although experimental designs were close between our study and the latter, discrepancies in the training sessions and the test selection criteria existed. Indeed Nawroth et al. [42] mainly selected animals on their ability to follow the experimenter’s arm holding a grape from one bowl to the other and strictly choosing the rewarded bowl which presented similarities with the pointing gestures. It may have impacted the results by facilitating the task. This will have to be confirmed by comparing performances of naive pigs (as in our study) and pigs used to seeing a human holding a reward (as in [42]).

Finally the domestic pigs we used in this experiment did not seem to share the spontaneous abilities of dogs to follow the human pointing gesture or the voice direction to find a hidden reward [16, 17, 19]. Therefore our results do not support the domestication hypothesis reported in dogs, which may be partly due to the different methods used [16]. Although domestication may have increased pigs’ capacities to communicate with humans, their everyday living experience may be poorer than that of dogs, restricting the extent to which these capacities are expressed in pigs. The role of early experience (i.e. early socialization in [13]) seems to be quite important for the development of the human-animal relationship, and this may impact the later interactive capacities of animals. Comparing performances of extensively handled piglets and puppies as well as less (or not) handled piglets and puppies would help to discriminate the effects of domestication from those of early experience.

Use of combined pointing gesture and human voice direction to find a hidden reward by pigs was possible in our experiment from the third test. Another mechanism which could affect piglets’ success could be a local enhancement created by the combination of signals (voice and pointing gesture, either dynamic or static). In both tests, the piglets’ attention could have been driven to the rewarding location by the signals. In certain conditions both pointing gestures can be used by piglets to find a hidden reward [42]. The local enhancement theory is sometimes argued in the comprehension of the pointing gesture in species expressing cooperative behaviour of benefit to conspecifics [13], such as [51] alarm barks or shared maternal care in pigs. These ideas need confirmation, including comparisons on the same task with other cooperative species. Yet, they still do not explain why several piglets were successful in the dynamic pointing and voice test but not in the subsequent static pointing and voice test.

Some piglets may have needed a clear demonstration of the positioning of the reward to succeed. Others may have needed more trials to understand the task. The first hypothesis is that the movement of the arm helped the piglets to understand the task they had to complete. Before we used dynamic pointing and voice, piglets had never seen the experimenter rewarding the containers, so they might have not understood that the experimenter was giving information about the rewarding location. Nawroth et al. [52] showed that piglets have limited abilities in mental representation of movements of hidden objects. They can use a signal to find a reward after seeing its displacement from one container to another but not when the reward is hidden. Showing piglets the rewarding process by using a dynamic pointing gesture might have helped them understand the test conditions. This may be important for some piglets, as eight out of ten piglets which succeeded in using the dynamic pointing gesture did not succeed in the next test when the reward was hidden again before the trial and the signal was no longer dynamic. A second hypothesis that may be complementary for certain piglets or possibly the only mechanism for others, is that the repetition of trials during the experiment helped the piglets to understand the task. We did not find learning patterns between test sessions, which corresponded to our expectation as we did not want piglets to learn but wanted to test their spontaneous behaviour. Nawroth and von Borell [45] reported that piglets needed up to 72 or 84 trials to reach the success criterion using visual or auditory signals respectively in the same sessions of a test. This large number of trials would be closer to a training process instead of simply testing pigs’ spontaneous inter-specific communicational capacities. In our study, piglets had a maximum of 32 trials in one test, but those that were involved in the first two tests had had 49 trials with static pointing and voice direction combination, which is substantially below 72. However, at the last test, piglets had already undergone 81 trials with pointing (static and dynamic) and voice. This is perhaps why some of them succeeded at this test. We could not test the improvement along trials or sessions between tests, as they combined differences in the signals and in the sessions’ organisation. If we combine these hypotheses, it would mean spontaneous use of pointing and voice direction is unlikely under our experimental/test conditions, and that some learning is necessary.

We wanted to determine if auditory signals alone could be sufficient for piglets to succeed. This was not the case. This means that piglets might have relied more on the pointing gestures than on the voice when the two signals were used in combination, however, this does not mean they do not have the capacity to use voice direction. As pigs preferentially rely on visual cues in their foraging behaviour [53]; their attention to the auditory signal might have been reduced when tested, as they were focusing on the visual information. Similar findings were observed in chimpanzees which were given a combination of gaze and vocalization signals to retrieve a hidden reward [27]. Emitting one signal after the other or both signals together did not seem to influence the results. The human vocalizations were considered more to be gathering attention to the situation than to the reward. Precautions are still needed as the pointing gestures were not tested alone at the end of our experiment, thus we cannot assume that pointing alone (after an extensive experience of combined visual and auditory signals) would be sufficient for piglets to succeed. Methodological points may also have interfered. Indeed, piglets could hear the recordings just before we opened the starting area (as the playback started 3s before for practical reasons). This might have compromised their ability to associate the voice with the task [54].

In our study only two piglets reached the success criterion both with the dynamic and the static combinations of signals. This result does not confirm those observed in dogs [13] or dolphins [23] which react similarly to both static and dynamic pointing gestures. Seabrook and Bartle [33] underlined the importance of individuality in the reactions of pigs to signals, some may be more sensitive to static signals and other to dynamic ones. This could explain why one piglet succeeded with only voice direction as a signal. This piglet may preferentially use sounds when communicating with humans. Identifying piglets which are sensitive to specific signals could facilitate working with the animals, by adapting human behaviour. Relying on pigs’ ability to transmit behaviours and information and learn from each other [55], understanding of human-given signals could thereafter be transferred to the other pigs.

Finally we found no spontaneous response to signals, so it is likely that attention mechanisms or learning capacities were involved in this process. The lack of a signal aimed at attracting the attention of the piglets to the experimenter before a session may have compromised the outcome of the test. Indeed it has been reported that in the same task, even in dogs, there is a much lower success rate when signals are provided without first obtaining the attention of the test subject and establishing eye-contact [56]. In our design, we made the assumption that the proximity of the rewarded bowl and the experimenter would promote the comprehension of pointing gesture. We cannot exclude the theory that the ability to learn to use the combination of signals may be a result of domestication in piglets [17], even if learning abilities are moderately heritable and variable among individuals [57]. Testing the hypothesis would require comparisons of the spontaneous and/or learning capacities of domestic pigs with their wild counterparts.

The assured impact of experience (trial repetition, movement of the arm) is also demonstrated clearly by the fact that previous signal exposure (signals+ group) modified the responses to subsequent tests (latencies to the first visit, stares) and seemed to accelerate familiarization to the test conditions. No further differences were found for latency to visit the first bowl between sessions after the second test (test B, pointing and voice test 1) for pre-exposed piglets, while it continued to decrease for naïve piglets not exposed to the situation (signals-). Pre-exposed piglets also stared more at the experimenter during the first static pointing gesture and voice test than during the following tests. Experience may explain these variations as pre-exposure sped up the trials and increased attention to the experimenter. The process we used to select piglets that could be exposed to the first test (number of visits to the bowl, no stress vocalizations, handling ease…) might also have had a causative role in the behavioural differences we observed. Selecting animals on their reactivity (stress reactions, difficulties to handle) in the test area might have created a reactivity bias [57] between the groups, with the non-exposed group being more likely to include animals which had difficulty adapting to the test conditions. Nonetheless, we did not find differences in side preference between them, even though it was one of the selection criteria. In addition, the success rate was similar therefore they had the same cognitive capacities.

Behavioural differences between both groups can be further explained by the delay of 13 days between the last day of familiarisation and the first presentation of a static combination of signals test. During this period, signals- piglets were not exposed to signals and test conditions. According to Waiblinger et al. [58], test conditions in terms of novelty of the environment and isolation of the social group can impact animal responses in a test. For signals- piglets, the unusual test conditions applied in the first presentation of a static combination of signals test contributed to the unstable latencies to the first visit of a bowl and the higher proportions of stares at the experimenter. Our methodology could thus be improved by more training trials for signals- piglets in order to better acclimatize them to the test conditions.

We identified behaviours associated with trial outcome success when piglets were submitted to the dynamic combination of signals test and the second presentation of the static combination of signals test. Piglets had better chances of success when using indirect trajectories or looking longer at the experimenter before choosing, which could reflect non-impulsive response behaviour. In the study of Nawroth et al. [59] impulsivity corresponded to a lower latency to respond to the task. This was linked to higher piglet success rates. Non-impulsivity would help the pigs to analyse the situation before the response. Due to our lack of successful piglets, we could not test the link between latencies and the success rate of each piglet, to make our results comparable to the ones of Nawroth. However, this preliminary result could be indicative of preferential behaviours associated with success in object-choice tasks. This could be complementary to latency of response proposed by Nawroth et al [59].In our setup, the chance of success in a trial was not linked to the latency to respond. Longer distances from the starting area or more numerous bowls could complicate the task and help identify other potential strategies, but this would apply only to designs with higher success rates.

Side preferences were present in the “previous signals exposure test”, however, piglets were specifically selected for their absence of side preference during training. This bias also appeared in other studies including goats [21] and piglets [42] and may not be the best indicator to recommend for selection criteria in experiments involving temperamental indicators. Balancing the positioning of the reward is thus an extremely important step in the design of this type of study as it appears difficult to prevent piglets from expressing side preferences.

## Conclusion

Piglets were able to use two combinations of visual and auditory signals to find a reward in an object-choice task. Individual success can be explained by two components: the repetition of the test situation including repeated exposure to the combination of signals and/or the change in the signal visual component. The use of the direction of voice by only one piglet in the last test supports the hypothesis of a preferential use of the visual component during exposure to the combinations of signals. Piglets staring at the experimenter and performing indirect trajectories increased their chances of success. Previous exposure to different signals familiarized piglets with testing conditions and stabilized their response more quickly in terms of latency of visit. Individual sensitivity to types of signals should be considered in future research. This would better identify clear interspecific signals in species which were not selected on their abilities to communicate through the same channels as us. The use of only one voice in this study excluded the potential impact of the physical characteristics of the human voice. The sensitivity of piglets to particular characteristics of the human voice needs more attention in further investigations.

## Supporting Information

S1 File Data set. (XLSX)
